# Microfluidics-Based Lab-on-Chip Systems in DNA-Based Biosensing: An Overview

**DOI:** 10.3390/s110605754

**Published:** 2011-05-27

**Authors:** Sabo Wada Dutse, Nor Azah Yusof

**Affiliations:** 1 Department of Chemistry, Faculty of Science, Universiti Putra Malaysia, 43400 UPM Serdang, Selangor, Malaysia; E-Mail: swdutse@yahoo.com; 2 Department of Science Laboratory Technology, Hussaini Adamu Federal Polytechnic, Kazaure, Nigeria

**Keywords:** microfluidics, lab on chip, environment

## Abstract

Microfluidics-based lab-on-chip (LOC) systems are an active research area that is revolutionising high-throughput sequencing for the fast, sensitive and accurate detection of a variety of pathogens. LOCs also serve as portable diagnostic tools. The devices provide optimum control of nanolitre volumes of fluids and integrate various bioassay operations that allow the devices to rapidly sense pathogenic threat agents for environmental monitoring. LOC systems, such as microfluidic biochips, offer advantages compared to conventional identification procedures that are tedious, expensive and time consuming. This paper aims to provide a broad overview of the need for devices that are easy to operate, sensitive, fast, portable and sufficiently reliable to be used as complementary tools for the control of pathogenic agents that damage the environment.

## 1. Introduction

Advances in microfluidics for nanotechnology-based sensing methods have been met with serious challenges in the creation of diagnostic devices that allow for the simultaneous detection of several types of biotargets on a single platform for environmental monitoring. The need to rapidly detect and characterise micro-organisms in environmental samples is imperative in many different industries, among which food and agriculture, healthcare, environmental monitoring, and biodefense are key players [1–3]. The inability to cultivate the majority of naturally occurring micro-organisms despite the demonstrated need necessitates a fast, sensitive and reliable platform, such as a microfluidics-based lab-on-chip (LOC) system. In the field of environmental monitoring, serious attention is needed in the evaluation of microbial cells in water, soil and the environment. A list of some biological threat agents compiled by the Centers for Disease Control and Prevention (CDC) in the United States [4] includes such notable agents as *Bacillus anthracis* (anthrax), *Francisella tularensis* (tularaemia), *Yersinia pestis* (plague), *Variola major* (smallpox), botulinum toxin (botulism), *Coxiella burnetii* (Q fever), *Brucella* spp. (brucellosis), *Vibrio cholera* (cholera), ricin, *Shigella* and *Salmonella* spp. These biological agents are transmitted via food, water, insect vectors, as aerosols or by direct contact (for extensive details, see [5–7]). The study of microorganism evolution and populations under conditions, such as during bio-waste composting, also requires highly sensitive devices [8]. Microfabrication technology has led to the miniaturisation of biosensors in response to increased demand for their use in environmental and medical diagnostic applications for environmental monitoring [9].

Global Industry Analysts, Inc. [10], have indicated that biosensors provide low-cost, compact, and low-power devices for environmental monitoring and point-of-care (POC) medical applications. Point-of-care testing (POCT), which is commonly described as bedside, near-patient, ancillary, and decentralised laboratory testing used for clinical diagnostics, is considered one of the main driving forces for the future in vitro diagnostic market [11]. The demand for dissolved-oxygen (DO) biosensors will continue to grow with increasingly poor water quality and the desire to preserve natural resources to maintain the health of people and the environment. The October 2001 anthrax attacks in the United States, outbreaks of severe acute respiratory syndrome (SARS), bovine spongiform encephalopathy (BSE, commonly known as mad-cow disease), Iraq’s acknowledgement following the Gulf War that it possessed loaded biological weapons, and many other threats and biological “incidents” worldwide have increased global demand for the tools to rapidly identify causative agents and infected individuals before the agents spread beyond control [12]. This need for detection necessitates the development of biodefense devices using a microfluidics approach to monitor and control food sources, water sources, and suspect powders, and to test for decontamination after the treatment of equipment, personnel, and key environments [13]. The advent of microfluidic chips has enabled the application of biosensors in warfare threat detection and security. Microfabrication and newer manufacturing techniques will continue to increase the number of applications for current biosensors in environmental monitoring and health care. The use of inexpensive, transistor-based biosensors has recently transformed the medical research field [14].

## 2. Microfluidics

Microfluidics traces its history from the microelectronics industry where researchers attempted to improve silicon-based micromachining processes using photolithography, etching, and bonding techniques. The first silicon-based analysis system was introduced in 1979 by Terry *et al*. Later, in the 1990s Manz *et al*. advanced the application of microfluidics [12].

**Figure 1.**
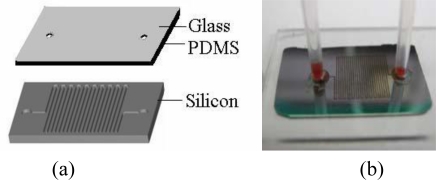
**(a)** Layout of microchip [21]; **(b)** Photograph of the microchip [21].

Whereas laboratory-scale samples are measured in millilitre-scale volumes [15], microfluidics involves the measurement of nanolitre- [12] and microlitre-scale volumes [16]. The term *microfluidics* therefore refers to any technology that moves microscopic and nanoscale volumes of fluid through micro-sized channels on a microelectromechanical system (MEMS). Microfluidics is a concept that combines the disciplines of fluid mechanics, surface science, chemistry, biology, and in many cases, optics, microscopy, electronics, control systems, and microfabrication [17]. Research in this field involves interdisciplinary integration. Programmable microfluidic chips, *i.e*., LOC systems, can automate biological computations or experiments by integrating a diverse set of biological sensors and manipulating fluids at the picolitre [18,19] and nanolitre scales [20]. Tian *et al.* first established a micro-solid-phase extraction (SPE) DNA purification system (Figure 1(a,b)) in a capillary packed with silica resin [21].

Health-care systems would greatly benefit from faster, more accurate and more highly precise diagnostic devices, such as microfluidics-based LOCs, which would significantly reduce health care costs while simultaneously providing better epidemiological data that can be used for infectious-disease modelling [22]. The microchip with a poly(dimethylsiloxane) (PDMS)-glass cover and a silicon substrate, shown in Figure 1(a,b), is 2 cm × 1.5 cm. The silicon substrate contains an etched coiled channel that is 25 cm long, 200 μm wide and 120 μm deep, and the cover includes two holes that are drilled in positions according to the silicon substrate [21]. The microfluidic chip shown in Figure 1(a,b) can be used for high-purity DNA extraction.

LOC-based pathogen sensors are competitive with laboratory-scale technologies in the analysis of complex biological samples. The analysis of a biological sample involves various processing steps, such as sample preparation, analyte enrichment, labelling, signal amplification and signal detection, that are performed on the chip. Therefore, only highly integrated micro-devices, or “micro total analysis systems” (μTAS), have real-world applications [23].

A variety of materials, including silicon, glass, soft or hard polymers and biomaterials (e.g., calcium alginate, cross-linked gelatine or hydrogels) have been used for microfabrication [24]. The choice of polymeric materials is often limited to solvent-resistant materials, such as Teflon, photopatternable silicon elastomers, thermoset polyesters, poly(methylmethacrylate) (PMMA) and patterned poly-(dimethylsiloxane) (PDMS), polyimide and SU-8 (negative photoresist) polymers [25,26].

## 3. The Physics of Microfluidics

**Figure 2.**
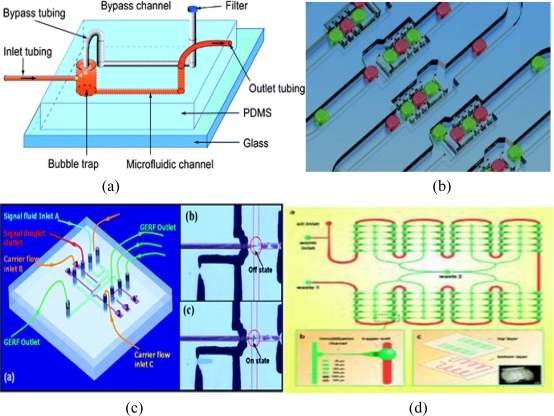
**(a)** Integrated bubble trap (IBT) [29]; **(b)** Microfluidic droplet-based shift register [30]; **(c)** Pure microfluid logic using a giant electro-rheological fluid as a working medium [31]; **(d)** Floatage-based droplet microfluidics [32].

An understanding of the underlying flow physics and interfacial phenomena at small scales is necessary when designing and optimising microfluidics-based devices for biological applications in environmental monitoring. A precise fluid control and flow stability are critical for successful DNA detection in microfluidics-based systems. Because microfluidic devices contain sensitive detection systems, the infusion of any fluids must be performed with the utmost care to prevent bubble formation within the channels or chambers. Although bubbles can be used as an actuation mechanism for various applications [27], the presence of undesired bubbles can adversely affect the sample flow and cause detection failures, particularly in highly sensitive optical detection schemes [3]. The manipulation of nanolitre to picolitre volumes of fluids on silicon chip surfaces has improved the chemical sensors’ microreactors, which has subsequently improved their detection limits. An eloquent review of flow physics in micro- and nano-scale fluidic devices is presented in the review article by Squires *et al*. [28]. Illustrations showing the micro-flow physics of some of the microfluidics channels designed and used by researchers are presented in Figure 2.

The design of a microfluidic device mostly depends on its target use. Figure 2 shows different designs, each representing an example of a design intended for a particular research area. The device shown in Figure 2(a) has been reported by the authors to have excellent properties: it is structurally simple, easily fabricated, does not interfere with the flow system, and is stable [29]. The microfluidic design is a PDMS microfluidic system fabricated for long-period cell cultures. An IBT helps prevent the accumulation of bubbles in the microfluidic channels that change the microenvironment of adherent cells and lead to cell extinction. Wenfu *et al*. [29] have reported that MC 3T3 E1 cells cultured in an IBT increased microfluidic channel yields and led to active proliferation after continuous flow for 10 days.

The device shown in Figure 2(b) enables the controllable serial formation, storage and retrieval of arrayed droplet networks in an automated, high-throughput manner. The microfluidic device was designed for the serial formation, storage and retrieval of water micro-droplets in oil. Its operation is analogous to that of an electronic shift register. Because droplets translate uniform information about their source, the droplets can be arrayed and serially shifted within the device. The principle of the device’s operation allows the controllable positioning of emulsions and the formation of interfaces between drops through the adjustment of the balance between hydrodynamic pressure and surface tension across a drop. The advantage of this system is that droplet networks are easily arrayed in a series of elements and cascaded within the channels to allow for investigations of dynamic biological processes based on molecular diffusion through the interfaces [30].

The device shown in Figure 2(c) represents a novel method for realising pure microfluidic logic with the help of a giant electro-rheological fluid (GERF) as the working medium. The microfluidic device contains logic-control components that incorporate a GERF with reversible characteristics through the liquid–solid phase transition in an external electric field. Four electrodes attached on the two microchannel sides act as the input and output signals of droplets: one pair controls the flow status, and the other pair detects signal generation. The logic consists of an IF gate and its inverter function is a NOT gate [31].

Figure 2(d) is also a novel floatage-based droplet microfluidic device for continuously characterising the neurotoxin-induced multiple responses in individual *Caenorhabditis elegans* (*C. elegans*). The microfluidic device was designed to simultaneously evaluate the movement and analyse the fluorescence imaging of individual *C. elegans*. Pharmacologists used the device to understand the mechanism leading to dopaminergic (DAergic) toxicity by neurotoxins and to screen new therapeutics for neurodegenerative diseases [32]. Most of the commercially available devices for POCT of proteins are lateral-flow assays, which are usually called immunochromatographic assays [11]. The continuous flow of liquid through micro-fabricated channels is inherently difficult to integrate because the parameters that control the flow field—pressure, fluid resistance, and electric-field strength—vary along the flow path [33,34]. Several characteristics of small-scale fluid flow involve laminar flow. Laminar flow creates easy flow patterns with very little diffusion (which eliminates potential difficulties in the mixing process), small volumes (which reduces the waste of expensive reagents), and easy fluid control with the help of pumps (which allows for the easy automation of fluid handling). The earliest micro-pump was developed by Smits in the 1980s. Later, in an attempt to improve the generated pressure, different pumping mechanisms were explored for chemical and biological applications, including thermopneumatics [35,36], electrostatics [37,38], piezoelectrics [39], electro-magnetics [40,41] and hydrogels [42].

In the past, hydrodynamic (electrokinetic) systems were most commonly used to control flow direction in an open-loop stream system of a microfluidic device. The concept was used to measure pressure differences in the microchannels and enable the realisation of a low-pressure manometer in microfluidic devices. The pressure technique solely depends on the flow-rate ratio of two fluids. This ratio can become too high when the fraction of one fluid becomes large. Electro-osmosis has recently become the preferred control technique because it has advantages over the pressure-driven method. Electro-osmosis gives a uniform flow-velocity profile and controls and guides sample streams in a multi-flow microfluidic system [43]. However, the electro-osmotic flow can become unstable when the voltage becomes too high. Currently, a combination of hydrodynamic and electro-osmosis methods are employed in microsystems [44,45] to avoid the problems of pressure-driven and electro-osmotic methods. In earlier methods, the flow-rate ratio or the electric fields were required to be manually adjusted because they were based on an open-loop control without feedback. Fluorescence detection [45] now allows for the return of a feedback signal to the flow-driven mechanisms. Combining pressure-driven methods, electro-osmotic effects and fluorescence detection produces a feedback signal for automatic control of the interface location via fluorescent intensity detection [45].

### 3.1. Dimensionless Numbers

Two basic dimensionless numbers are important in the context of fluid mechanics and species transport: the Reynolds number (Re) and the Peclet number (Pe). The Reynolds number is the most important of the dimensionless numbers in force flows because it dictates whether the flow is laminar or turbulent [46]. The Reynolds number is the ratio of inertia to viscous force densities and can be determined from the following equation:

(1) Re = ρνDh/μ

where *ρ* is the density of the fluid, *ν* is the characteristic velocity of the fluid, *D_h_* is the hydraulic diameter of the channel and *μ* is the viscosity of the fluid. In the micro-flow regime, the Re ranges from 10^−6^ to 10.

The dimensionless number that characterises the nature and strength of the diffusive mixing is referred to as the Peclet number (Pe). The Pe represents the relative strength of convection *versus* diffusion and can be determined from:

(2) Pe = vw/D

where *w* is the width of the microchannel, *υ* is the characteristic velocity of the fluid and *D* is the diffusion coefficient of the solute particles. Because of the ineffectiveness of diffusion-dependent mixing, researchers have developed other innovative strategies for mixing by secondary or transverse flow. Unavoidable shear flow and diffusion in the microchannels makes inter-sample and dead volumes difficult to eliminate [47].

Other noteworthy dimensionless numbers in specific appliances include the Knudsen number (*Kn*), which signifies the ratio of the molecular mean free path with the characteristic system length scale; the capillary number (*Ca*), which represents the ratio between the viscous and surface tension forces; the Weissenberg number (*Wi*), which is the ratio between the relaxation time and the shear rate of polymers; and the Deborah number (*De*), which represents the ratio of the polymer relaxation time to the characteristic flow time. For further reference, detailed discussions on these numbers are available elsewhere [28].

### 3.2. Droplet Flow

Researchers are shifting from traditional continuous-flow-based systems in microfluidics research to droplet-flow-based systems referred to as digital microfluidics. The circulation flow within the droplet and the high surface-to-volume ratio enhances efficient mixing and provides thermal dissipation with short reaction times [47]. The interfacial stress balance between a droplet and the continuous phase at its interface is preserved [48]:

(3) $$\begin{array}{l} (\tau_{d}\;-\;\tau_{a})\;nt\;-\;\nabla y\;t\;=\;0\\ (p_{d}\;-\;p_{a})\;-\;\gamma \nabla n\;\;=\;0 \end{array}\}$$

where ***τ*** is the deviatoric stress tensor, ***n*** is the unit normal of the interface pointing out of the droplet, ***t*** is a tangential vector of unit length at the interface, *p* is hydrodynamic pressure, *γ* is the interfacial tension coefficient and *∇* is the interfacial divergence; the subscripts *_d_* and *_a_* denote properties of the droplet and the ambient continuous phase, respectively.

In most electrically controlled digital microfluidic devices [47,49], the droplets are either conductive or highly polarisable. At the droplet surface:

(4) $$\begin{array}{l} n\;(-{ɛ}_{a,i}\;\nabla \phi_{a,i})\;=\;\sigma \\ \frac{\delta \sigma }{\delta t}\;+\;\nabla_{\Sigma }\;\sigma v\;+\;n\;k\;\nabla \phi_{d}\;=\;0 \end{array}\}$$

where *σ* is the surface charge density at the droplet surface, *v* is the fluid velocity inside the droplet, *k* is the droplet conductivity, *∇_Σ_* is the interfacial divergence, ϕ is the electric potential and ɛ is the electric permittivity; the subscripts *_a_,* *_d_* and *_i_* denote the continuous phase, droplets and contacting solid phase, respectively.

## 4. Microfluidics-Based Pathogen Detection

Microfluidic biochips for pathogen sensing have been applied to microarray technology. The detection of DNA hybridisation is obtained through a variety of different electrochemical techniques, including electroactive hybridisation indicators, enzymes, and nanoparticles [3]. An integrated microfluidic microarray technology has allowed the identification of fungal pathogens [50–55]. With enhanced MEMS technology, it is feasible to incorporate all the functional components of a macro-scale instrument into the restricted spatial domains of a microchannel system [46]. The manipulation required for electrochemical DNA detection begins with the immobilisation of an ssDNA capture probe on an electrode surface. After the probe has been immobilised, baseline electrical measurements are performed; then the target DNA is added and is allowed to hybridise with the captured DNA, after which another set of electrical measurements is performed to detect the electrode changes resulting from DNA hybridisation. The detection of the DNA can be improved by modification of the DNA with electroactive compounds or metallic nanoparticles.

Wang *et al*. have reported that the detection capability of DNA and RNA sequences is becoming more important for the diagnosis of diseases [56] and for the detection of pathogenic organisms, such as *Escherichia coli* [57], *Bacillus anthracis* [58], *Cryptosporidium parvum* [59] and dengue virus [60]. High-throughput systems for the rapid detection of nucleic acids that identify specific bacterial pathogens have been reported [61]. Target amplification techniques represent a prominent and commonly applied method of accurately detecting small amounts of infectious pathogens. The amplifications that lead to higher sensitivity can be achieved through polymerase chain reaction (PCR), ligase chain reaction (LCR) or nucleic-acid-sequence-based amplification (NASBA) [62]. A major setback with LOC devices is the unspecific adsorption resulting from the large surface-to-volume ratios that exist in the microchannels [63], which inhibits the PCR reaction.

Environmental pathogens often exist in food and water; these pathogens include bacteria, viruses, parasites and toxins [64]. Prior to the advancement of nanotechnology, medical professionals had difficulty detecting any case of biopathogenic outbreak before a report of symptoms from an infected host (plant or animal). The infection of a host (*i.e*., a human) can lead to quarantine to limit further transmission of the disease, depending on the pathogen type. The pathogenic threat to humans and the environment has necessitated quick biopathogen detection and identification [65] for better monitoring. Microfluidics-based LOCs can serve this purpose.

The traditional western blotting method for detection is time consuming, and its requirement of highly skilled technicians exacerbates a challenging situation. The efficiency and high performance that result from the small sample volumes and rapid response times of microfluidic devices have enabled their penetration into nearly all corners of the life sciences [66]. These advantages also render the devices capable of the sensitive DNA-based detection of pathogens in environmental samples. Among a number of nanotechnology-based diagnostic systems, microfluidics-based LOC systems play a significant role in environmental microbial monitoring because they detect and identify targets within minutes with a single-cell sensitivity level [67–69]. The miniaturisation of biopathogenic detectors into a POC system opens significant opportunities for environmental monitoring, taking into consideration the system’s portability, good precision, disposability, automation, rapid measurement capabilities and low sample consumption. However, the use of small sample volumes requires that the samples be concentrated to achieve the required sensitivity.

### DNA-Based Biosensor

An electrochemical detection system for DNA sensing can be achieved by the catalysed reduction and oxidation of DNA bases or through the electrochemical response displayed by redox markers in a specific binding event with the target DNA. An integrated microfluidic electrochemical DNA (IMED) sensor has performed three main biochemical functions: symmetric polymerase chain reaction (PCR), enzymatic single-stranded DNA (ssDNA) generation, and sequence-specific electrochemical detection [70]. Electrochemical DNA hybridisation biosensors are based on the ability of ssDNA to match with its counterpart strand of a complementary nucleotide sequence [71], as well as washing steps [72,73] and the immobilisation of the capture probe on the electrode. Nanoparticles, nanogold [74,75], and zirconia [76] have been used to modify the electrode for DNA-probe (ssDNA) immobilisation. Fractal analysis [77] has been used to analyse the hybridisation of different targets (400 nM) in solution to a probe immobilised on the DNA chip surface [78], the hybridisation of various concentrations in nanomoles (nM) of free-DNA in solution to 22-mer strand (bond DNA) immobilised through a phenylene-diisocyanate linker molecule on a glass substrate [79], and the binding (hybridisation) of a complimentary and a non-complimentary (three-base mismatch strand) DNA in a solution to a 30-mer 3′-thiolated DNA strand immobilised on an electrochemical enzymatic genosensor [80]. Fractal analysis may provide a good option for the kinetic analysis of the binding and dissociation of hybridisation in analyte-receptor reactions performed on biosensor surfaces [77].

The interactions of redox complexes with DNA in solution have been studied through cyclic voltammetry. Voltammetric peak currents generally decrease with the decreased mass diffusion of DNA that results from its binding to metal complexes, and the level of signal reduction is a function of DNA concentration [81]. These effects form the basis for real-time DNA detection. Various methods of enhancing electrochemical DNA biosensor sensitivity using redox markers have been reported. Electroactive redox markers that intercalate their targets produce chemical signals and are mostly products of polymers [73,82]; metal complexes, such as cadmium complex [83]; organic dyes [84]; and ruthenium complex and its derivatives [81,85].

The extraction of DNA from a sample serves as a useful method for retrieving genetic information about its source. The extractions of DNA from soil samples, especially forest soil, are occasionally contaminated by humic substances. These substances create interferences that arise from their similar chemical and physical characteristics to soil. Researchers have developed a number of strategies to eliminate such contaminants. During the stages of DNA extraction, for example, electrophoresis has been carried out in different pH buffers to eliminate the interfering effects [86]. Recent soil genetic research includes the cry gene resources of *Bacillus thuringiensis* in soil [87], soil bacteria community composition by 16S rRNA gene clones [88], and the diversity of diazotrophic bacteria in peat soil through the cloning of the nifH gene [89].

## 5. Conclusions

We have presented an overview of the research on microfluidics-based LOC systems for DNA-based biosensors. We first provided an overview of the threat posed by pathogenic micro-organisms that affect the environment, and the new challenges introduced by the advent of microfluidics were highlighted. The fluid mechanics of the systems at the nanoscale were then discussed. The inherent challenges of fluid flow in microchannels were analysed, although issues of using mechanical pumps for fluid transmission still require attention. Basic research in fluid mechanics and the transport phenomena of fluids greater than nanolitre-scale volumes, along with technological advancements in the fabrication and control processes, will indeed play significant roles in the automation of fluid flow in microchannels. The main application of microfluidics in pathogen detection involves DNA-based methods through electrochemical techniques [70,72]. Various methods of electrode modification are needed to improve the results, and the choice of electrode depends on the approach of the researcher; details of this issue were also highlighted in this paper. The environmental threats by pathogenic micro-organisms are real because plants and animals are suffering from the consequences. There is a clear need for devices, such as microfluidics-based LOCs in controlling the effects of pathogens in the environment.
